# Crystal structure of 2-[(*E*)-4-benz­yloxy-2-hy­droxy­benzyl­idene]-*N*-cyclo­hexyl­hydrazinecarbo­thio­amide aceto­nitrile hemisolvate

**DOI:** 10.1107/S1600536814017905

**Published:** 2014-08-09

**Authors:** N. R. Sajitha, M. Sithambaresan, M. R. Prathapachandra Kurup

**Affiliations:** a Department of Applied Chemistry, Cochin University of Science and Technology, Kochi 682 022, India; b Department of Chemistry, Faculty of Science, Eastern University, Sri Lanka, Chenkalady, Sri Lanka

**Keywords:** crystal structure, hydrazinecarbo­thio­amide, hydrogen bonding, C—H⋯π inter­actions, anti­microbial applications

## Abstract

The asymmetric unit of the title compound, C_21_H_25_N_3_O_2_S·0.5C_2_H_3_N, contains two independent mol­ecules with almost similar structural properties along with a solvent mol­ecule of aceto­nitrile. The compound exists in the *E* conformation with respect to the azomethine C=N double bond. The hydrazinecarbo­thio­amide moieties in both independent mol­ecules are almost planar [maximum deviations of 0.013 (2) and 0.007 (2) Å]. The mol­ecular conformation is stabilized in each case by an intra­molecular N—H⋯N hydrogen bond. In the crystal, pairs of N—H⋯S hydrogen bonds link each of the independent mol­ecules into inversion dimers. The dimers are inter­connected by means of three C—H⋯π inter­actions.

## Related literature   

For anti­microbial application, see: Joseph *et al.* (2004 ▶). For fluorescence activity, see: Kumar *et al.* (2013 ▶). For versatile coordination ability, see: Sreekanth *et al.* (2004 ▶). For the synthesis of related compounds, see: Jacob & Kurup (2012 ▶). For related structures, see: Seena *et al.* (2006 ▶); Jacob & Kurup (2012 ▶). 
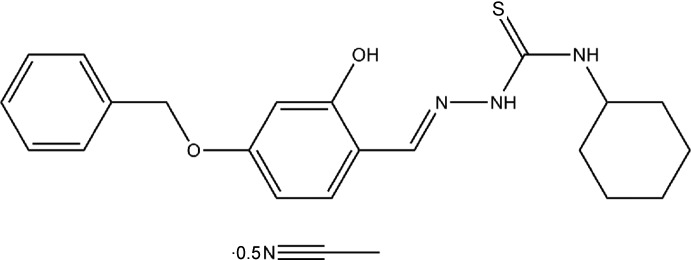


## Experimental   

### Crystal data  

2C_21_H_25_N_3_O_2_S·C_2_H_3_N
*M* *_r_* = 808.07
Triclinic, 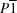

*a* = 10.5345 (4) Å
*b* = 10.8341 (4) Å
*c* = 21.8169 (10) Å
α = 97.241 (2)°
β = 92.120 (2)°
γ = 118.901 (2)°
*V* = 2148.72 (15) Å^3^
*Z* = 2
Mo *K*α radiation
μ = 0.17 mm^−1^
*T* = 296 K
0.50 × 0.20 × 0.18 mm

### Data collection  

Bruker Kappa APEXII CCD diffractometer
Absorption correction: multi-scan *SADABS* (Bruker, 2004 ▶) *T*_min_ = 0.918, *T*_max_ = 0.942
15933 measured reflections
9260 independent reflections
6918 reflections with *I* > 2σ(*I*)
*R*_int_ = 0.018

### Refinement  

*R*[*F* ^2^ > 2σ(*F* ^2^)] = 0.046
*wR*(*F* ^2^) = 0.136
*S* = 1.03
9260 reflections
540 parameters
6 restraints
H atoms treated by a mixture of independent and constrained refinement
Δρ_max_ = 0.39 e Å^−3^
Δρ_min_ = −0.25 e Å^−3^

### 

Data collection: *APEX2* (Bruker, 2004 ▶); cell refinement: *APEX2* and *SAINT* (Bruker, 2004 ▶); data reduction: *SAINT* and *XPREP* (Bruker, 2004 ▶); program(s) used to solve structure: *SHELXS97* (Sheldrick, 2008 ▶); program(s) used to refine structure: *SHELXL97* (Sheldrick, 2008 ▶); molecular graphics: *ORTEP-3 for Windows* (Farrugia, 2012 ▶) and *DIAMOND* (Brandenburg, 2010 ▶); software used to prepare material for publication: *SHELXL97* and *publCIF* (Westrip, 2010 ▶).

## Supplementary Material

Crystal structure: contains datablock(s) Global, I. DOI: 10.1107/S1600536814017905/bv2235sup1.cif

Structure factors: contains datablock(s) I. DOI: 10.1107/S1600536814017905/bv2235Isup2.hkl

Supporting information file. DOI: 10.1107/S1600536814017905/bv2235Isup3.cml

**Fig. 1.**
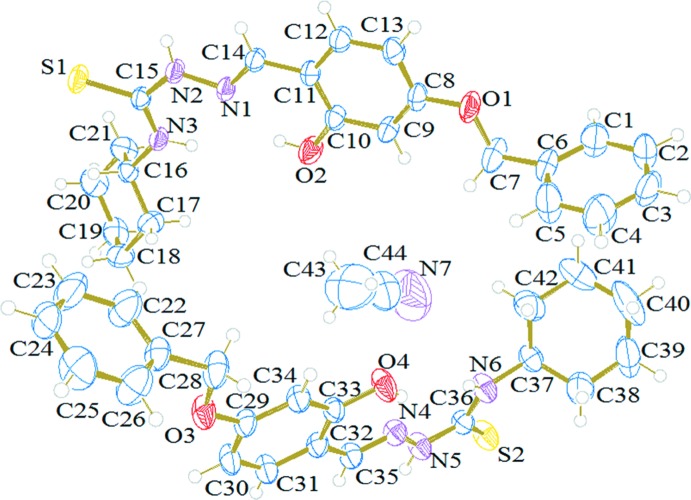
ORTEP diagram of (E)-2-(4-benzyloxy-2-hydroxybenzylidene)-N-cyclohexylhydrazinecarbothioamide with 50% probability ellipsoids.

ORTEP E N . DOI: 10.1107/S1600536814017905/bv2235fig1.tif
*ORTEP* diagram of (*E*)-2-(4-benz­yloxy-2-hy­droxy­benzyl­idene)-*N*-cyclo­hexyl­hydrazinecarbo­thio­amide with 50% probability ellipsoids.

**Fig. 2.**
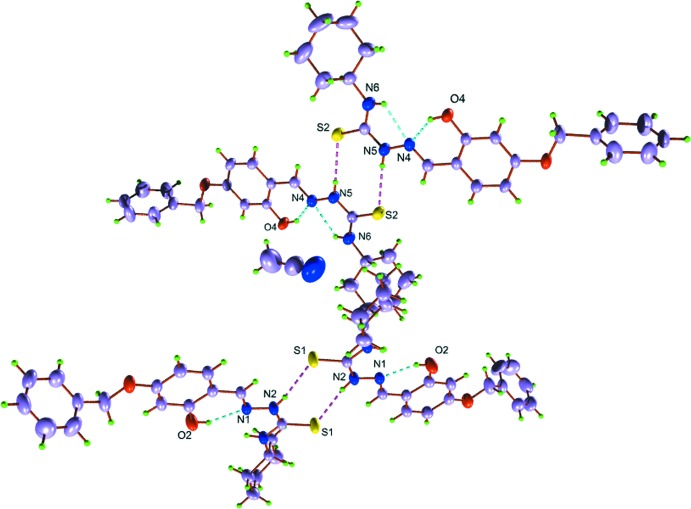
Hydrogen-bond interactions of the title compound, [C21H25N3O2S]·0.5C2H3N.

21 25 3 2 2 3 . DOI: 10.1107/S1600536814017905/bv2235fig2.tif
Hydrogen-bond inter­actions of the title compound, [C_21_H_25_N_3_O_2_S]·0.5C_2_H_3_N.

**Fig. 3.**
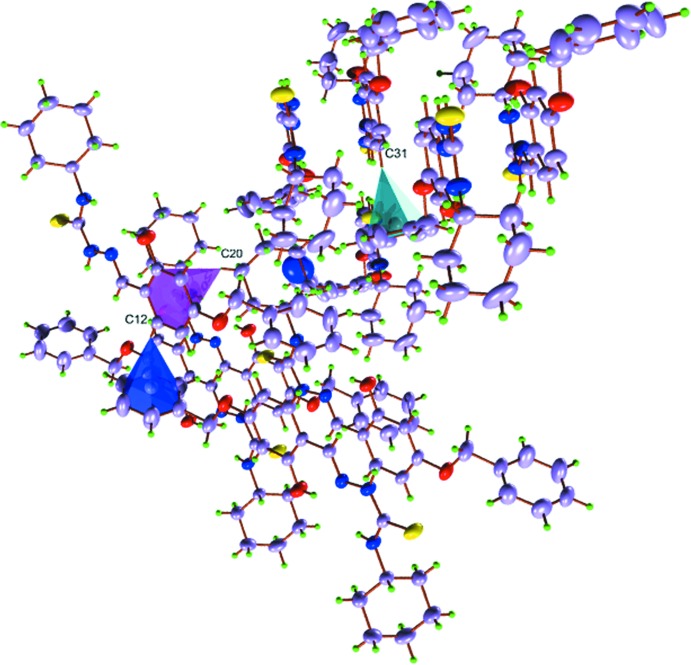
C—H···π interactions of the title compound.

**Fig. 4.**
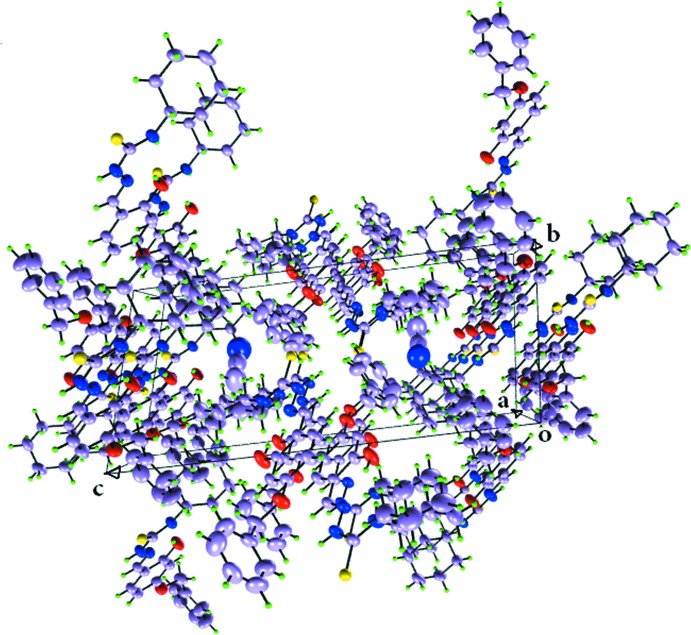
Packing diagram of the title compound along a axis.

. DOI: 10.1107/S1600536814017905/bv2235fig3.tif
C—H⋯π inter­actions of the title compound.

a . DOI: 10.1107/S1600536814017905/bv2235fig4.tif
Packing diagram of the title compound along *a* axis.

CCDC reference: 1017712

Additional supporting information:  crystallographic information; 3D view; checkCIF report

## Figures and Tables

**Table 1:** Hydrogen-bond geometry (Å, °)

*D*—H⋯*A*	*D*—H	H⋯*A*	*D*⋯*A*	*D*—H⋯*A*
N5—H5′⋯S2^i^	0.88 (1)	2.48 (1)	3.3495 (17)	173 (2)
N2—H2′⋯S1^ii^	0.87 (1)	2.44 (1)	3.3047 (16)	171 (2)
O2—H2*A*⋯N1	0.84 (1)	1.96 (2)	2.696 (2)	146 (3)
O4—H4′⋯N4	0.84 (1)	1.94 (2)	2.680 (2)	146 (2)
C12—H12⋯*Cg*1^iii^	0.93	2.95	3.811 (2)	154
C20—H20*B*⋯*Cg*2^iv^	0.96	2.87	3.715 (2)	146
C31—H31⋯*Cg*4^v^	0.93	2.84	3.714 (2)	157

Symmetry codes: (i) 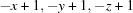; (ii) 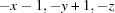; (iii) 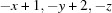; (iv) 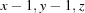; (v) 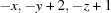.

## supplementary crystallographic information

### S1. Comment

Thiosemicarbazones are important class of compounds due to their antimicrobial activity (Joseph *et al.*, 2004). They are also found to act as turn on fluorescent sensors for fluoride anion (Kumar *et al.*, 2013). These compounds are important due to their ability to show versatile coordination abilities in complexes (Sreekanth *et al.*, 2004). Here we present a new N4-substituted thioemicarbazone with interesting structural properties.

The asymmetric unit of the title compound consists of two independent molecules of the thiosemicarbazone and one acetonitrile molecule giving an overall ratio of thiosemicarbazone to acetonitrile to 2:1. The compound crystallizes into triclinic space group P-1. The geometric parameters of each molecule are almost identical. The molecules adopt *E* configuration with respect to C14—N1 and C35—N4 bonds which is confirmed by the C14/N1/N2/C15 and C35/N4/N5/C36 torsion angles of -177.55 (17)° and 175.50 (18)° respectively (Fig.1). The N1/N2/C15/S1 and N4/N5/C36/S2 torsion angles of 171.78 (3)° and -173.54 (4)° suggest that the thionyl atom S1 of the first molecule and S2 of the second molecule are located *trans* to azomethine nitrogens N1 and N4 respectively. The torsion angles -6.0 (3)° and 5.2 (3)° for N1/N2/C15/N3 and N4/N5/C36/N6 respectively confirm the *cis* configuration of N1 with respect to N3 and N4 with respect to N6.

The C14—N1 and C35—N4 bond distances [1.281 (2) and 1.278 (2) Å] are close to that of formal C═N bond [1.284 (3) Å] (Seena *et al.*, 2006). Similarly the C15—S1 and C36—S2 bond distances [1.6835 (17) Å and 1.6824 (17) Å] are also close to that of formal C═S bond [1.68 (3) Å] (Jacob & Kurup, 2012). The hydrazine carbothioamide moieties in both molecules are almost planar with maximum deviation of 0.013 (2) Å for atom C15 and 0.007 (2) Å for C36 respectively from their least square planes. The cyclohexyl rings in both the molecules adopt chair conformation. The least square plane calculations show that the rings C1–C6/C8–C13 in one molecule and C22–C27/C29–C34 in the other molecule are twisted with a dihedral angles of 82.93 (120)° and 88.59 (12)° respectively.

Whilst one the molecules in the asymmetric unit has only one intramolecular hydrogen bond of the type O—H···N with D···A distance 2.696 (2) Å and one N—H···S type intermolecular hydrogen bonding interaction with D···A distance 3.3046 (18) Å, the other molecule has two types of intramolecular hydrogen bonds of O—H···N and N—H···N with D···A distances 2.680 (2) and 2.655 (3) Å respectively along with one type of N—H···S intermolecular hydrogen bonding with D···A distance 3.3495 (18) Å (Table 1). All these intermolecular interactions present in two asymmetric molecules build two centrosymmetric dimers in the crystal lattice (Fig 2). These dimers are interconnected by means of three C—H···π interactions with H···π distances of 2.95, 2.87 and 2.84 Å (Fig. 3). Fig 4 shows the packing of molecules along crystallographic *a* axis.

### S2. Experimental

The preparation of the compound involves a two step process (Jacob & Kurup, 2012). In the first step, cyclohexylisothiocyanate (15 mmol, 2 ml) in 15 ml methanol and hydrazine hydrate (90 mmol, 4.3 ml) in 15 ml methanol were mixed and the resulting solution was stirred for an hour when the colourless product, *N*(4)-cyclohexylthiosemicarbazide formed was filtered, washed with methanol and dried *in vacuo*. In the second step, 4-benzyloxy-2-hydroxybenzaldehyde (0.2283 g, 1 mmol), dissolved in 15 ml acetonitrile was added to a solution of *N*(4)-cyclohexylthiosemicarbazide in 10 ml acetonitrile and the reaction mixture was refluxed for 3 hrs in acidic medium. The resultant solution was kept for one week to give yellow crystals of the compound (yield 51.59%, 0.1978 g)

IR (KBr, ν in cm^-1^): 3400, 3130, 2985, 2929, 2851, 1627, 1599, 1540, 1505, 1224.

### S3. Refinement

All H atoms on C were placed in calculated positions, guided by difference maps, with C—H bond distances of 0.93 Å. H atoms were assigned *U*_iso_(H) values of 1.2Ueq (carrier). H atoms of N—H bonds were located from difference maps and the bond distances are restrained to 0.88±0.01 Å. Omitted owing to bad disagreement was (0 0 2). The phenolic H atoms were located from difference maps and the O–H bond distances were restrained to 0.84±0.01 Å.

### Crystal data

**Table:**

2C_21_H_25_N_3_O_2_S·C_2_H_3_N	*Z* = 2
*M**_r_* = 808.07	*F*(000) = 860
Triclinic, *P*1	*D*_x_ = 1.249 Mg m^−^^3^
Hall symbol: -P 1	Mo *K*α radiation, λ = 0.71073 Å
*a* = 10.5345 (4) Å	Cell parameters from 6744 reflections
*b* = 10.8341 (4) Å	θ = 2.2–28.1°
*c* = 21.8169 (10) Å	µ = 0.17 mm^−^^1^
α = 97.241 (2)°	*T* = 296 K
β = 92.120 (2)°	Needle, yellow
γ = 118.901 (2)°	0.50 × 0.20 × 0.18 mm
*V* = 2148.72 (15) Å^3^	

### Data collection

**Table:**

Bruker Kappa APEXII CCD diffractometer	9260 independent reflections
Radiation source: fine-focus sealed tube	6918 reflections with *I* > 2σ(*I*)
Graphite monochromator	*R*_int_ = 0.018
ω and φ scan	θ_max_ = 27.0°, θ_min_ = 1.0°
Absorption correction: multi-scan *SADABS* (Bruker, 2004)	*h* = −13→13
*T*_min_ = 0.918, *T*_max_ = 0.942	*k* = −13→13
15933 measured reflections	*l* = −27→19

### Refinement

**Table:**

Refinement on *F*^2^	Secondary atom site location: difference Fourier map
Least-squares matrix: full	Hydrogen site location: inferred from neighbouring sites
*R*[*F*^2^ > 2σ(*F*^2^)] = 0.046	H atoms treated by a mixture of independent and constrained refinement
*wR*(*F*^2^) = 0.136	*w* = 1/[σ^2^(*F*_o_^2^) + (0.0652*P*)^2^ + 0.7212*P*] where *P* = (*F*_o_^2^ + 2*F*_c_^2^)/3
*S* = 1.03	(Δ/σ)_max_ < 0.001
9260 reflections	Δρ_max_ = 0.39 e Å^−^^3^
540 parameters	Δρ_min_ = −0.25 e Å^−^^3^
6 restraints	Extinction correction: *SHELXL97* (Sheldrick, 2008), Fc^*^=kFc[1+0.001xFc^2^λ^3^/sin(2θ)]^-1/4^
Primary atom site location: structure-invariant direct methods	Extinction coefficient: 0.0036 (9)

### Special details

**Table:**

Geometry. All e.s.d.'s (except the e.s.d. in the dihedral angle between two l.s. planes) are estimated using the full covariance matrix. The cell e.s.d.'s are taken into account individually in the estimation of e.s.d.'s in distances, angles and torsion angles; correlations between e.s.d.'s in cell parameters are only used when they are defined by crystal symmetry. An approximate (isotropic) treatment of cell e.s.d.'s is used for estimating e.s.d.'s involving l.s. planes.
Refinement. Refinement of *F*^2^ against ALL reflections. The weighted *R*-factor *wR* and goodness of fit *S* are based on *F*^2^, conventional *R*-factors *R* are based on *F*, with *F* set to zero for negative *F*^2^. The threshold expression of *F*^2^ > σ(*F*^2^) is used only for calculating *R*-factors(gt) *etc*. and is not relevant to the choice of reflections for refinement. *R*-factors based on *F*^2^ are statistically about twice as large as those based on *F*, and *R*- factors based on ALL data will be even larger.

### Fractional atomic coordinates and isotropic or equivalent isotropic displacement parameters (Å^2^)

**Table:**

	*x*	*y*	*z*	*U*_iso_*/*U*_eq_	
S1	−0.60227 (5)	0.39508 (6)	0.07407 (3)	0.05080 (16)	
S2	0.62422 (6)	0.49312 (6)	0.42617 (3)	0.05093 (16)	
O1	0.50839 (13)	0.84763 (15)	0.05748 (7)	0.0500 (4)	
O2	0.04715 (15)	0.53001 (16)	0.11875 (8)	0.0542 (4)	
O3	0.15464 (15)	1.13678 (15)	0.44214 (7)	0.0484 (4)	
O4	0.48815 (17)	0.97664 (18)	0.38472 (8)	0.0576 (4)	
N1	−0.18643 (14)	0.53120 (15)	0.06462 (7)	0.0339 (3)	
N2	−0.33002 (15)	0.49724 (17)	0.05214 (8)	0.0380 (4)	
N3	−0.39713 (15)	0.35494 (16)	0.12662 (7)	0.0364 (3)	
N4	0.47289 (17)	0.75978 (16)	0.43719 (7)	0.0378 (3)	
N5	0.51039 (18)	0.65922 (17)	0.44969 (8)	0.0420 (4)	
N6	0.65106 (19)	0.70656 (17)	0.36985 (8)	0.0451 (4)	
N7	0.3302 (6)	0.4151 (5)	0.2682 (2)	0.1717 (19)	
C1	0.8362 (2)	0.9162 (3)	0.07453 (12)	0.0616 (6)	
H1	0.8023	0.8397	0.0420	0.074*	
C2	0.9830 (3)	1.0168 (3)	0.08441 (14)	0.0701 (7)	
H2	1.0472	1.0058	0.0591	0.084*	
C3	1.0339 (2)	1.1302 (3)	0.12998 (14)	0.0702 (7)	
H3	1.1325	1.1982	0.1359	0.084*	
C4	0.9401 (3)	1.1445 (3)	0.16734 (16)	0.0886 (10)	
H4	0.9744	1.2227	0.1991	0.106*	
C5	0.7939 (3)	1.0434 (3)	0.15839 (14)	0.0759 (8)	
H5	0.7307	1.0541	0.1844	0.091*	
C6	0.74039 (19)	0.9283 (2)	0.11217 (11)	0.0467 (5)	
C7	0.5819 (2)	0.8176 (2)	0.10456 (13)	0.0579 (6)	
H7A	0.5705	0.7231	0.0928	0.070*	
H7B	0.5409	0.8202	0.1435	0.070*	
C8	0.35980 (18)	0.78021 (19)	0.05220 (9)	0.0384 (4)	
C9	0.27479 (18)	0.67962 (19)	0.08791 (9)	0.0396 (4)	
H9	0.3177	0.6486	0.1158	0.048*	
C10	0.12462 (18)	0.62556 (18)	0.08164 (9)	0.0357 (4)	
C11	0.05770 (17)	0.66738 (17)	0.03884 (9)	0.0333 (4)	
C12	0.14739 (19)	0.76540 (19)	0.00217 (9)	0.0390 (4)	
H12	0.1049	0.7930	−0.0275	0.047*	
C13	0.29580 (19)	0.8219 (2)	0.00862 (10)	0.0416 (4)	
H13	0.3530	0.8876	−0.0160	0.050*	
C14	−0.09743 (17)	0.61556 (18)	0.03108 (9)	0.0340 (4)	
H14	−0.1347	0.6449	0.0002	0.041*	
C15	−0.43444 (17)	0.41394 (17)	0.08528 (8)	0.0327 (4)	
C16	−0.49581 (18)	0.26337 (18)	0.16725 (8)	0.0345 (4)	
H16	−0.5634	0.2984	0.1783	0.041*	
C17	−0.4086 (2)	0.2746 (2)	0.22629 (10)	0.0483 (5)	
H17A	−0.3572	0.3731	0.2473	0.058*	
H17B	−0.3367	0.2461	0.2161	0.058*	
C18	−0.5082 (2)	0.1797 (2)	0.26935 (10)	0.0549 (5)	
H18A	−0.4495	0.1831	0.3055	0.066*	
H18B	−0.5719	0.2160	0.2836	0.066*	
C19	−0.5994 (3)	0.0272 (2)	0.23745 (12)	0.0593 (6)	
H19A	−0.6665	−0.0283	0.2652	0.071*	
H19B	−0.5365	−0.0131	0.2280	0.071*	
C20	−0.6847 (2)	0.0171 (2)	0.17822 (12)	0.0580 (6)	
H20A	−0.7556	0.0473	0.1881	0.070*	
H20B	−0.7374	−0.0814	0.1574	0.070*	
C21	−0.5842 (2)	0.1104 (2)	0.13481 (10)	0.0487 (5)	
H21A	−0.5191	0.0749	0.1218	0.058*	
H21B	−0.6419	0.1059	0.0980	0.058*	
C22	−0.0343 (3)	1.1977 (3)	0.34536 (13)	0.0700 (7)	
H22	−0.0480	1.1139	0.3215	0.084*	
C23	−0.1360 (3)	1.2417 (3)	0.33712 (15)	0.0828 (9)	
H23	−0.2180	1.1869	0.3083	0.099*	
C24	−0.1165 (3)	1.3645 (3)	0.37091 (13)	0.0654 (7)	
H24	−0.1835	1.3956	0.3645	0.078*	
C25	0.0003 (3)	1.4413 (3)	0.41389 (14)	0.0728 (7)	
H25	0.0134	1.5251	0.4375	0.087*	
C26	0.1011 (3)	1.3960 (2)	0.42301 (14)	0.0663 (7)	
H26	0.1802	1.4488	0.4534	0.080*	
C27	0.0858 (2)	1.2751 (2)	0.38802 (10)	0.0445 (5)	
C28	0.1956 (2)	1.2258 (2)	0.39536 (12)	0.0550 (6)	
H28A	0.1967	1.1726	0.3563	0.066*	
H28B	0.2923	1.3073	0.4075	0.066*	
C29	0.22107 (19)	1.05710 (19)	0.44817 (9)	0.0371 (4)	
C30	0.1700 (2)	0.9661 (2)	0.49167 (10)	0.0444 (5)	
H30	0.0989	0.9654	0.5159	0.053*	
C31	0.2259 (2)	0.8774 (2)	0.49842 (10)	0.0420 (4)	
H31	0.1922	0.8171	0.5279	0.050*	
C32	0.33155 (19)	0.87403 (18)	0.46276 (9)	0.0342 (4)	
C33	0.38348 (19)	0.96924 (19)	0.42039 (9)	0.0363 (4)	
C34	0.3293 (2)	1.0614 (2)	0.41346 (9)	0.0387 (4)	
H34	0.3658	1.1255	0.3856	0.046*	
C35	0.38347 (19)	0.77485 (18)	0.47119 (9)	0.0373 (4)	
H35	0.3504	0.7199	0.5026	0.045*	
C36	0.5958 (2)	0.62855 (19)	0.41394 (9)	0.0365 (4)	
C37	0.7455 (2)	0.6898 (2)	0.32651 (9)	0.0426 (4)	
H37	0.7157	0.5879	0.3177	0.051*	
C38	0.9025 (3)	0.7690 (3)	0.35401 (13)	0.0663 (7)	
H38A	0.9126	0.7339	0.3916	0.080*	
H38B	0.9340	0.8700	0.3650	0.080*	
C39	0.9981 (3)	0.7489 (4)	0.30791 (17)	0.0894 (10)	
H39A	0.9722	0.6490	0.2998	0.107*	
H39B	1.0995	0.8041	0.3256	0.107*	
C40	0.9792 (4)	0.7970 (3)	0.24691 (17)	0.0951 (11)	
H40A	1.0133	0.8989	0.2544	0.114*	
H40B	1.0376	0.7793	0.2174	0.114*	
C41	0.8222 (4)	0.7181 (3)	0.22016 (13)	0.0812 (9)	
H41A	0.7916	0.6174	0.2086	0.097*	
H41B	0.8117	0.7539	0.1828	0.097*	
C42	0.7237 (3)	0.7347 (3)	0.26621 (12)	0.0688 (7)	
H42A	0.7459	0.8336	0.2740	0.083*	
H42B	0.6225	0.6764	0.2486	0.083*	
C43	0.2358 (7)	0.5888 (6)	0.2730 (3)	0.170 (2)	
H43A	0.2010	0.5951	0.3127	0.256*	
H43B	0.1575	0.5572	0.2405	0.256*	
H43C	0.3136	0.6810	0.2682	0.256*	
C44	0.2868 (4)	0.4920 (5)	0.2694 (2)	0.1059 (11)	
H3'	−0.3084 (13)	0.368 (2)	0.1287 (10)	0.047 (6)*	
H6	0.631 (2)	0.7759 (16)	0.3704 (10)	0.046 (6)*	
H5'	0.473 (2)	0.611 (2)	0.4799 (8)	0.051 (6)*	
H2'	−0.349 (2)	0.533 (2)	0.0218 (8)	0.057 (7)*	
H2A	−0.0407 (13)	0.508 (3)	0.1130 (13)	0.078 (9)*	
H4'	0.516 (3)	0.920 (2)	0.3942 (12)	0.064 (7)*	

### Atomic displacement parameters (Å^2^)

**Table:**

	*U*^11^	*U*^22^	*U*^33^	*U*^12^	*U*^13^	*U*^23^
S1	0.0261 (2)	0.0718 (3)	0.0646 (4)	0.0249 (2)	0.0140 (2)	0.0391 (3)
S2	0.0694 (4)	0.0562 (3)	0.0588 (3)	0.0499 (3)	0.0283 (3)	0.0274 (3)
O1	0.0206 (6)	0.0553 (8)	0.0659 (10)	0.0111 (6)	0.0027 (6)	0.0155 (7)
O2	0.0293 (7)	0.0642 (9)	0.0646 (10)	0.0139 (7)	0.0056 (7)	0.0353 (8)
O3	0.0501 (8)	0.0593 (8)	0.0634 (9)	0.0430 (7)	0.0211 (7)	0.0294 (7)
O4	0.0688 (10)	0.0879 (11)	0.0613 (10)	0.0645 (9)	0.0364 (8)	0.0435 (9)
N1	0.0213 (6)	0.0393 (8)	0.0391 (8)	0.0130 (6)	0.0025 (6)	0.0082 (6)
N2	0.0224 (7)	0.0496 (9)	0.0448 (9)	0.0164 (6)	0.0067 (6)	0.0218 (7)
N3	0.0230 (7)	0.0445 (8)	0.0432 (9)	0.0151 (6)	0.0057 (6)	0.0182 (7)
N4	0.0445 (8)	0.0399 (8)	0.0437 (9)	0.0305 (7)	0.0082 (7)	0.0130 (7)
N5	0.0544 (10)	0.0471 (9)	0.0483 (10)	0.0393 (8)	0.0204 (8)	0.0224 (8)
N6	0.0562 (10)	0.0451 (9)	0.0538 (10)	0.0361 (8)	0.0229 (8)	0.0221 (8)
N7	0.191 (5)	0.199 (5)	0.184 (5)	0.141 (4)	0.050 (4)	0.026 (4)
C1	0.0497 (13)	0.0629 (14)	0.0688 (16)	0.0271 (11)	0.0060 (11)	0.0017 (12)
C2	0.0409 (12)	0.097 (2)	0.0799 (19)	0.0362 (13)	0.0195 (12)	0.0247 (16)
C3	0.0278 (10)	0.0832 (18)	0.0795 (19)	0.0097 (11)	−0.0039 (11)	0.0250 (15)
C4	0.0506 (15)	0.0773 (18)	0.093 (2)	0.0053 (13)	−0.0019 (14)	−0.0199 (16)
C5	0.0414 (12)	0.0736 (16)	0.093 (2)	0.0185 (12)	0.0145 (13)	−0.0135 (14)
C6	0.0256 (9)	0.0454 (10)	0.0662 (14)	0.0157 (8)	−0.0020 (9)	0.0095 (10)
C7	0.0294 (10)	0.0527 (12)	0.0854 (17)	0.0143 (9)	−0.0028 (10)	0.0183 (12)
C8	0.0220 (8)	0.0373 (9)	0.0488 (11)	0.0103 (7)	0.0027 (7)	0.0017 (8)
C9	0.0264 (8)	0.0402 (9)	0.0490 (11)	0.0142 (7)	−0.0014 (8)	0.0079 (8)
C10	0.0264 (8)	0.0336 (8)	0.0425 (10)	0.0107 (7)	0.0035 (7)	0.0079 (7)
C11	0.0233 (8)	0.0326 (8)	0.0402 (10)	0.0110 (7)	0.0034 (7)	0.0046 (7)
C12	0.0292 (9)	0.0412 (9)	0.0452 (11)	0.0149 (8)	0.0034 (8)	0.0124 (8)
C13	0.0281 (9)	0.0410 (10)	0.0490 (11)	0.0102 (8)	0.0083 (8)	0.0131 (8)
C14	0.0255 (8)	0.0366 (9)	0.0387 (10)	0.0137 (7)	0.0022 (7)	0.0091 (7)
C15	0.0242 (8)	0.0348 (8)	0.0380 (10)	0.0129 (7)	0.0041 (7)	0.0092 (7)
C16	0.0297 (8)	0.0363 (9)	0.0383 (10)	0.0150 (7)	0.0075 (7)	0.0128 (7)
C17	0.0415 (10)	0.0488 (11)	0.0472 (12)	0.0149 (9)	0.0008 (9)	0.0158 (9)
C18	0.0548 (13)	0.0676 (14)	0.0455 (12)	0.0284 (11)	0.0095 (10)	0.0270 (11)
C19	0.0565 (13)	0.0598 (13)	0.0741 (16)	0.0309 (11)	0.0267 (12)	0.0380 (12)
C20	0.0491 (12)	0.0432 (11)	0.0670 (15)	0.0092 (9)	0.0145 (11)	0.0160 (10)
C21	0.0474 (11)	0.0409 (10)	0.0459 (12)	0.0123 (9)	0.0067 (9)	0.0072 (9)
C22	0.0744 (16)	0.0748 (16)	0.0774 (18)	0.0561 (14)	−0.0123 (14)	−0.0097 (13)
C23	0.0769 (18)	0.112 (2)	0.080 (2)	0.0692 (18)	−0.0205 (15)	−0.0063 (17)
C24	0.0754 (16)	0.0935 (18)	0.0693 (16)	0.0695 (16)	0.0170 (14)	0.0320 (14)
C25	0.0910 (19)	0.0588 (14)	0.091 (2)	0.0547 (15)	0.0133 (17)	0.0093 (14)
C26	0.0603 (14)	0.0538 (13)	0.0848 (19)	0.0312 (12)	−0.0106 (13)	0.0033 (12)
C27	0.0422 (10)	0.0472 (10)	0.0598 (13)	0.0303 (9)	0.0115 (9)	0.0236 (10)
C28	0.0467 (11)	0.0628 (13)	0.0794 (16)	0.0383 (11)	0.0190 (11)	0.0393 (12)
C29	0.0362 (9)	0.0419 (9)	0.0457 (11)	0.0275 (8)	0.0064 (8)	0.0131 (8)
C30	0.0419 (10)	0.0558 (11)	0.0542 (12)	0.0347 (9)	0.0208 (9)	0.0218 (9)
C31	0.0424 (10)	0.0477 (10)	0.0506 (12)	0.0292 (9)	0.0182 (9)	0.0238 (9)
C32	0.0348 (9)	0.0364 (9)	0.0398 (10)	0.0227 (7)	0.0063 (7)	0.0112 (7)
C33	0.0370 (9)	0.0469 (10)	0.0375 (10)	0.0282 (8)	0.0103 (8)	0.0148 (8)
C34	0.0406 (10)	0.0464 (10)	0.0435 (11)	0.0289 (8)	0.0117 (8)	0.0214 (8)
C35	0.0405 (9)	0.0381 (9)	0.0429 (10)	0.0247 (8)	0.0098 (8)	0.0144 (8)
C36	0.0409 (9)	0.0384 (9)	0.0394 (10)	0.0259 (8)	0.0072 (8)	0.0094 (8)
C37	0.0469 (11)	0.0385 (9)	0.0473 (11)	0.0228 (8)	0.0161 (9)	0.0127 (8)
C38	0.0500 (13)	0.0692 (15)	0.0706 (17)	0.0236 (12)	0.0104 (12)	0.0043 (13)
C39	0.0498 (14)	0.095 (2)	0.114 (3)	0.0298 (15)	0.0298 (16)	0.0049 (19)
C40	0.090 (2)	0.0655 (16)	0.116 (3)	0.0219 (16)	0.072 (2)	0.0228 (17)
C41	0.108 (2)	0.0781 (18)	0.0607 (17)	0.0425 (17)	0.0419 (16)	0.0292 (14)
C42	0.0760 (17)	0.0828 (17)	0.0606 (15)	0.0430 (14)	0.0248 (13)	0.0340 (13)
C43	0.236 (6)	0.213 (6)	0.147 (5)	0.177 (5)	0.008 (4)	0.031 (4)
C44	0.092 (2)	0.127 (3)	0.106 (3)	0.060 (2)	0.021 (2)	0.011 (2)

### Geometric parameters (Å, º)

**Table:**

S1—C15	1.6834 (17)	C18—H18A	0.9700
S2—C36	1.6824 (17)	C18—H18B	0.9700
O1—C8	1.363 (2)	C19—C20	1.508 (3)
O1—C7	1.424 (3)	C19—H19A	0.9700
O2—C10	1.355 (2)	C19—H19B	0.9700
O2—H2A	0.838 (10)	C20—C21	1.524 (3)
O3—C29	1.3631 (19)	C20—H20A	0.9700
O3—C28	1.427 (2)	C20—H20B	0.9700
O4—C33	1.350 (2)	C21—H21A	0.9700
O4—H4'	0.843 (10)	C21—H21B	0.9700
N1—C14	1.281 (2)	C22—C27	1.364 (3)
N1—N2	1.3798 (19)	C22—C23	1.382 (3)
N2—C15	1.346 (2)	C22—H22	0.9300
N2—H2'	0.872 (9)	C23—C24	1.353 (4)
N3—C15	1.318 (2)	C23—H23	0.9300
N3—C16	1.464 (2)	C24—C25	1.347 (4)
N3—H3'	0.873 (9)	C24—H24	0.9300
N4—C35	1.278 (2)	C25—C26	1.385 (3)
N4—N5	1.3786 (19)	C25—H25	0.9300
N5—C36	1.343 (2)	C26—C27	1.364 (3)
N5—H5'	0.875 (9)	C26—H26	0.9300
N6—C36	1.323 (2)	C27—C28	1.501 (2)
N6—C37	1.455 (2)	C28—H28A	0.9700
N6—H6	0.875 (9)	C28—H28B	0.9700
N7—C44	1.125 (5)	C29—C34	1.378 (2)
C1—C6	1.368 (3)	C29—C30	1.387 (3)
C1—C2	1.383 (3)	C30—C31	1.366 (2)
C1—H1	0.9300	C30—H30	0.9300
C2—C3	1.342 (4)	C31—C32	1.394 (2)
C2—H2	0.9300	C31—H31	0.9300
C3—C4	1.357 (4)	C32—C33	1.396 (2)
C3—H3	0.9300	C32—C35	1.448 (2)
C4—C5	1.380 (3)	C33—C34	1.388 (2)
C4—H4	0.9300	C34—H34	0.9300
C5—C6	1.361 (3)	C35—H35	0.9300
C5—H5	0.9300	C37—C38	1.503 (3)
C6—C7	1.499 (3)	C37—C42	1.506 (3)
C7—H7A	0.9700	C37—H37	0.9800
C7—H7B	0.9700	C38—C39	1.518 (4)
C8—C13	1.385 (3)	C38—H38A	0.9700
C8—C9	1.385 (3)	C38—H38B	0.9700
C9—C10	1.390 (2)	C39—C40	1.529 (5)
C9—H9	0.9300	C39—H39A	0.9700
C10—C11	1.393 (2)	C39—H39B	0.9700
C11—C12	1.400 (2)	C40—C41	1.498 (5)
C11—C14	1.443 (2)	C40—H40A	0.9700
C12—C13	1.370 (2)	C40—H40B	0.9700
C12—H12	0.9300	C41—C42	1.527 (4)
C13—H13	0.9300	C41—H41A	0.9700
C14—H14	0.9300	C41—H41B	0.9700
C16—C21	1.509 (3)	C42—H42A	0.9700
C16—C17	1.511 (3)	C42—H42B	0.9700
C16—H16	0.9800	C43—C44	1.386 (6)
C17—C18	1.521 (3)	C43—H43A	0.9600
C17—H17A	0.9700	C43—H43B	0.9600
C17—H17B	0.9700	C43—H43C	0.9600
C18—C19	1.505 (3)		
			
C8—O1—C7	118.05 (16)	C16—C21—H21A	109.6
C10—O2—H2A	109.1 (19)	C20—C21—H21A	109.6
C29—O3—C28	118.03 (15)	C16—C21—H21B	109.6
C33—O4—H4'	108.7 (17)	C20—C21—H21B	109.6
C14—N1—N2	115.26 (14)	H21A—C21—H21B	108.1
C15—N2—N1	121.70 (15)	C27—C22—C23	121.0 (2)
C15—N2—H2'	122.2 (15)	C27—C22—H22	119.5
N1—N2—H2'	116.1 (15)	C23—C22—H22	119.5
C15—N3—C16	124.67 (14)	C24—C23—C22	120.2 (3)
C15—N3—H3'	117.7 (14)	C24—C23—H23	119.9
C16—N3—H3'	117.6 (14)	C22—C23—H23	119.9
C35—N4—N5	116.27 (15)	C25—C24—C23	119.6 (2)
C36—N5—N4	120.82 (15)	C25—C24—H24	120.2
C36—N5—H5'	120.2 (15)	C23—C24—H24	120.2
N4—N5—H5'	118.9 (15)	C24—C25—C26	120.3 (2)
C36—N6—C37	125.36 (15)	C24—C25—H25	119.9
C36—N6—H6	113.2 (14)	C26—C25—H25	119.9
C37—N6—H6	121.3 (14)	C27—C26—C25	120.9 (2)
C6—C1—C2	120.6 (2)	C27—C26—H26	119.5
C6—C1—H1	119.7	C25—C26—H26	119.5
C2—C1—H1	119.7	C26—C27—C22	117.92 (19)
C3—C2—C1	120.8 (2)	C26—C27—C28	122.2 (2)
C3—C2—H2	119.6	C22—C27—C28	119.9 (2)
C1—C2—H2	119.6	O3—C28—C27	107.81 (16)
C2—C3—C4	119.3 (2)	O3—C28—H28A	110.1
C2—C3—H3	120.3	C27—C28—H28A	110.1
C4—C3—H3	120.3	O3—C28—H28B	110.1
C3—C4—C5	120.2 (3)	C27—C28—H28B	110.1
C3—C4—H4	119.9	H28A—C28—H28B	108.5
C5—C4—H4	119.9	O3—C29—C34	123.98 (16)
C6—C5—C4	121.2 (2)	O3—C29—C30	115.26 (16)
C6—C5—H5	119.4	C34—C29—C30	120.75 (15)
C4—C5—H5	119.4	C31—C30—C29	118.94 (17)
C5—C6—C1	117.9 (2)	C31—C30—H30	120.5
C5—C6—C7	120.2 (2)	C29—C30—H30	120.5
C1—C6—C7	121.9 (2)	C30—C31—C32	122.41 (17)
O1—C7—C6	108.31 (17)	C30—C31—H31	118.8
O1—C7—H7A	110.0	C32—C31—H31	118.8
C6—C7—H7A	110.0	C31—C32—C33	117.39 (15)
O1—C7—H7B	110.0	C31—C32—C35	119.59 (16)
C6—C7—H7B	110.0	C33—C32—C35	123.01 (16)
H7A—C7—H7B	108.4	O4—C33—C34	117.05 (16)
O1—C8—C13	115.07 (16)	O4—C33—C32	121.98 (15)
O1—C8—C9	124.33 (17)	C34—C33—C32	120.97 (16)
C13—C8—C9	120.58 (15)	C29—C34—C33	119.46 (16)
C8—C9—C10	119.27 (17)	C29—C34—H34	120.3
C8—C9—H9	120.4	C33—C34—H34	120.3
C10—C9—H9	120.4	N4—C35—C32	122.63 (16)
O2—C10—C9	116.81 (16)	N4—C35—H35	118.7
O2—C10—C11	121.93 (15)	C32—C35—H35	118.7
C9—C10—C11	121.26 (16)	N6—C36—N5	117.15 (15)
C10—C11—C12	117.46 (15)	N6—C36—S2	123.67 (14)
C10—C11—C14	123.34 (16)	N5—C36—S2	119.17 (14)
C12—C11—C14	119.19 (16)	N6—C37—C38	111.66 (18)
C13—C12—C11	122.03 (17)	N6—C37—C42	109.76 (17)
C13—C12—H12	119.0	C38—C37—C42	112.18 (19)
C11—C12—H12	119.0	N6—C37—H37	107.7
C12—C13—C8	119.33 (17)	C38—C37—H37	107.7
C12—C13—H13	120.3	C42—C37—H37	107.7
C8—C13—H13	120.3	C37—C38—C39	110.4 (2)
N1—C14—C11	123.32 (16)	C37—C38—H38A	109.6
N1—C14—H14	118.3	C39—C38—H38A	109.6
C11—C14—H14	118.3	C37—C38—H38B	109.6
N3—C15—N2	117.35 (15)	C39—C38—H38B	109.6
N3—C15—S1	123.54 (13)	H38A—C38—H38B	108.1
N2—C15—S1	119.07 (13)	C38—C39—C40	110.8 (3)
N3—C16—C21	111.38 (15)	C38—C39—H39A	109.5
N3—C16—C17	109.39 (14)	C40—C39—H39A	109.5
C21—C16—C17	111.35 (16)	C38—C39—H39B	109.5
N3—C16—H16	108.2	C40—C39—H39B	109.5
C21—C16—H16	108.2	H39A—C39—H39B	108.1
C17—C16—H16	108.2	C41—C40—C39	110.5 (2)
C16—C17—C18	110.61 (16)	C41—C40—H40A	109.5
C16—C17—H17A	109.5	C39—C40—H40A	109.5
C18—C17—H17A	109.5	C41—C40—H40B	109.5
C16—C17—H17B	109.5	C39—C40—H40B	109.5
C18—C17—H17B	109.5	H40A—C40—H40B	108.1
H17A—C17—H17B	108.1	C40—C41—C42	111.9 (3)
C19—C18—C17	111.81 (19)	C40—C41—H41A	109.2
C19—C18—H18A	109.3	C42—C41—H41A	109.2
C17—C18—H18A	109.3	C40—C41—H41B	109.2
C19—C18—H18B	109.3	C42—C41—H41B	109.2
C17—C18—H18B	109.3	H41A—C41—H41B	107.9
H18A—C18—H18B	107.9	C37—C42—C41	110.5 (2)
C18—C19—C20	111.37 (17)	C37—C42—H42A	109.5
C18—C19—H19A	109.4	C41—C42—H42A	109.5
C20—C19—H19A	109.4	C37—C42—H42B	109.5
C18—C19—H19B	109.4	C41—C42—H42B	109.5
C20—C19—H19B	109.4	H42A—C42—H42B	108.1
H19A—C19—H19B	108.0	C44—C43—H43A	109.5
C19—C20—C21	111.10 (18)	C44—C43—H43B	109.5
C19—C20—H20A	109.4	H43A—C43—H43B	109.5
C21—C20—H20A	109.4	C44—C43—H43C	109.5
C19—C20—H20B	109.4	H43A—C43—H43C	109.5
C21—C20—H20B	109.4	H43B—C43—H43C	109.5
H20A—C20—H20B	108.0	N7—C44—C43	177.9 (6)
C16—C21—C20	110.36 (17)		
			
C14—N1—N2—C15	−177.55 (17)	C19—C20—C21—C16	−56.4 (2)
C35—N4—N5—C36	175.50 (18)	C27—C22—C23—C24	1.0 (5)
C6—C1—C2—C3	−1.8 (4)	C22—C23—C24—C25	−1.9 (5)
C1—C2—C3—C4	1.1 (4)	C23—C24—C25—C26	0.8 (4)
C2—C3—C4—C5	0.0 (5)	C24—C25—C26—C27	1.4 (4)
C3—C4—C5—C6	−0.3 (5)	C25—C26—C27—C22	−2.3 (4)
C4—C5—C6—C1	−0.3 (4)	C25—C26—C27—C28	178.0 (2)
C4—C5—C6—C7	178.2 (3)	C23—C22—C27—C26	1.1 (4)
C2—C1—C6—C5	1.3 (4)	C23—C22—C27—C28	−179.1 (3)
C2—C1—C6—C7	−177.2 (2)	C29—O3—C28—C27	167.25 (18)
C8—O1—C7—C6	−166.00 (18)	C26—C27—C28—O3	88.5 (3)
C5—C6—C7—O1	97.6 (3)	C22—C27—C28—O3	−91.3 (3)
C1—C6—C7—O1	−83.9 (3)	C28—O3—C29—C34	2.6 (3)
C7—O1—C8—C13	175.50 (19)	C28—O3—C29—C30	−176.06 (19)
C7—O1—C8—C9	−2.8 (3)	O3—C29—C30—C31	176.84 (19)
O1—C8—C9—C10	175.28 (18)	C34—C29—C30—C31	−1.9 (3)
C13—C8—C9—C10	−3.0 (3)	C29—C30—C31—C32	−0.6 (3)
C8—C9—C10—O2	−178.48 (17)	C30—C31—C32—C33	2.1 (3)
C8—C9—C10—C11	2.0 (3)	C30—C31—C32—C35	−178.45 (19)
O2—C10—C11—C12	−179.23 (17)	C31—C32—C33—O4	178.46 (19)
C9—C10—C11—C12	0.3 (3)	C35—C32—C33—O4	−1.0 (3)
O2—C10—C11—C14	1.2 (3)	C31—C32—C33—C34	−1.3 (3)
C9—C10—C11—C14	−179.29 (17)	C35—C32—C33—C34	179.32 (18)
C10—C11—C12—C13	−1.7 (3)	O3—C29—C34—C33	−175.91 (18)
C14—C11—C12—C13	177.95 (18)	C30—C29—C34—C33	2.7 (3)
C11—C12—C13—C8	0.7 (3)	O4—C33—C34—C29	179.20 (18)
O1—C8—C13—C12	−176.74 (17)	C32—C33—C34—C29	−1.1 (3)
C9—C8—C13—C12	1.7 (3)	N5—N4—C35—C32	−178.66 (17)
N2—N1—C14—C11	177.59 (16)	C31—C32—C35—N4	175.26 (19)
C10—C11—C14—N1	3.0 (3)	C33—C32—C35—N4	−5.3 (3)
C12—C11—C14—N1	−176.58 (17)	C37—N6—C36—N5	−179.92 (19)
C16—N3—C15—N2	179.03 (16)	C37—N6—C36—S2	−1.2 (3)
C16—N3—C15—S1	1.3 (3)	N4—N5—C36—N6	5.2 (3)
N1—N2—C15—N3	−6.0 (3)	N4—N5—C36—S2	−173.54 (14)
N1—N2—C15—S1	171.78 (13)	C36—N6—C37—C38	−84.5 (3)
C15—N3—C16—C21	84.1 (2)	C36—N6—C37—C42	150.4 (2)
C15—N3—C16—C17	−152.42 (18)	N6—C37—C38—C39	179.7 (2)
N3—C16—C17—C18	−179.61 (17)	C42—C37—C38—C39	−56.6 (3)
C21—C16—C17—C18	−56.1 (2)	C37—C38—C39—C40	56.6 (3)
C16—C17—C18—C19	54.8 (2)	C38—C39—C40—C41	−56.6 (3)
C17—C18—C19—C20	−54.8 (3)	C39—C40—C41—C42	55.7 (3)
C18—C19—C20—C21	55.5 (3)	N6—C37—C42—C41	179.9 (2)
N3—C16—C21—C20	179.42 (17)	C38—C37—C42—C41	55.1 (3)
C17—C16—C21—C20	57.0 (2)	C40—C41—C42—C37	−54.9 (3)

### Hydrogen-bond geometry (Å, º)

**Table:**

*D*—H···*A*	*D*—H	H···*A*	*D*···*A*	*D*—H···*A*
N6—H6···N4	0.88 (1)	2.21 (2)	2.655 (2)	111 (2)
N5—H5′···S2^i^	0.88 (1)	2.48 (1)	3.3495 (17)	173 (2)
N2—H2′···S1^ii^	0.87 (1)	2.44 (1)	3.3047 (16)	171 (2)
O2—H2*A*···N1	0.84 (1)	1.96 (2)	2.696 (2)	146 (3)
O4—H4′···N4	0.84 (1)	1.94 (2)	2.680 (2)	146 (2)
C12—H12···*Cg*1^iii^	0.93	2.95	3.811 (2)	154
C20—H20*B*···*Cg*2^iv^	0.96	2.87	3.715 (2)	146
C31—H31···*Cg*4^v^	0.93	2.84	3.714 (2)	157

Symmetry codes: (i) −*x*+1, −*y*+1, −*z*+1; (ii) −*x*−1, −*y*+1, −*z*; (iii) −*x*+1, −*y*+2, −*z*; (iv) *x*−1, *y*−1, *z*; (v) −*x*, −*y*+2, −*z*+1.
