# Supplementary figures and images for: Crystal structure of 2-[(E)-4-benz­yloxy-2-hy­droxy­benzyl­idene]-N-cyclo­hexyl­hydrazinecarbo­thio­amide aceto­nitrile hemisolvate
Source: Acta Crystallogr Sect E Struct Rep Online. 2014 Aug 9;70(Pt 9):o987–8. doi: 10.1107/S1600536814017905 (PMC4186196; doi:10.1107/S1600536814017905)

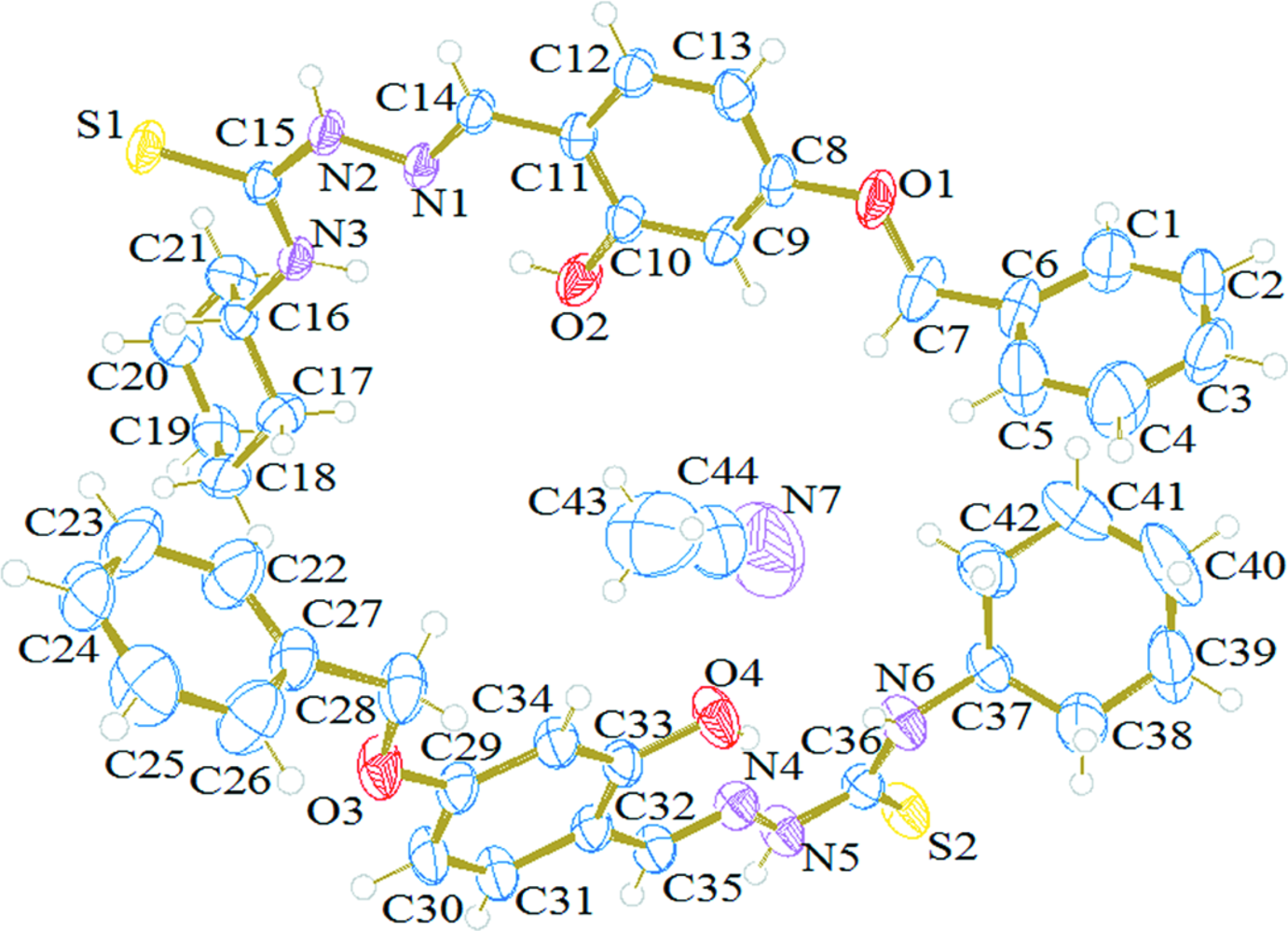

Supplement: Supplementary file 4 [file e-70-0o987-fig1.tif]

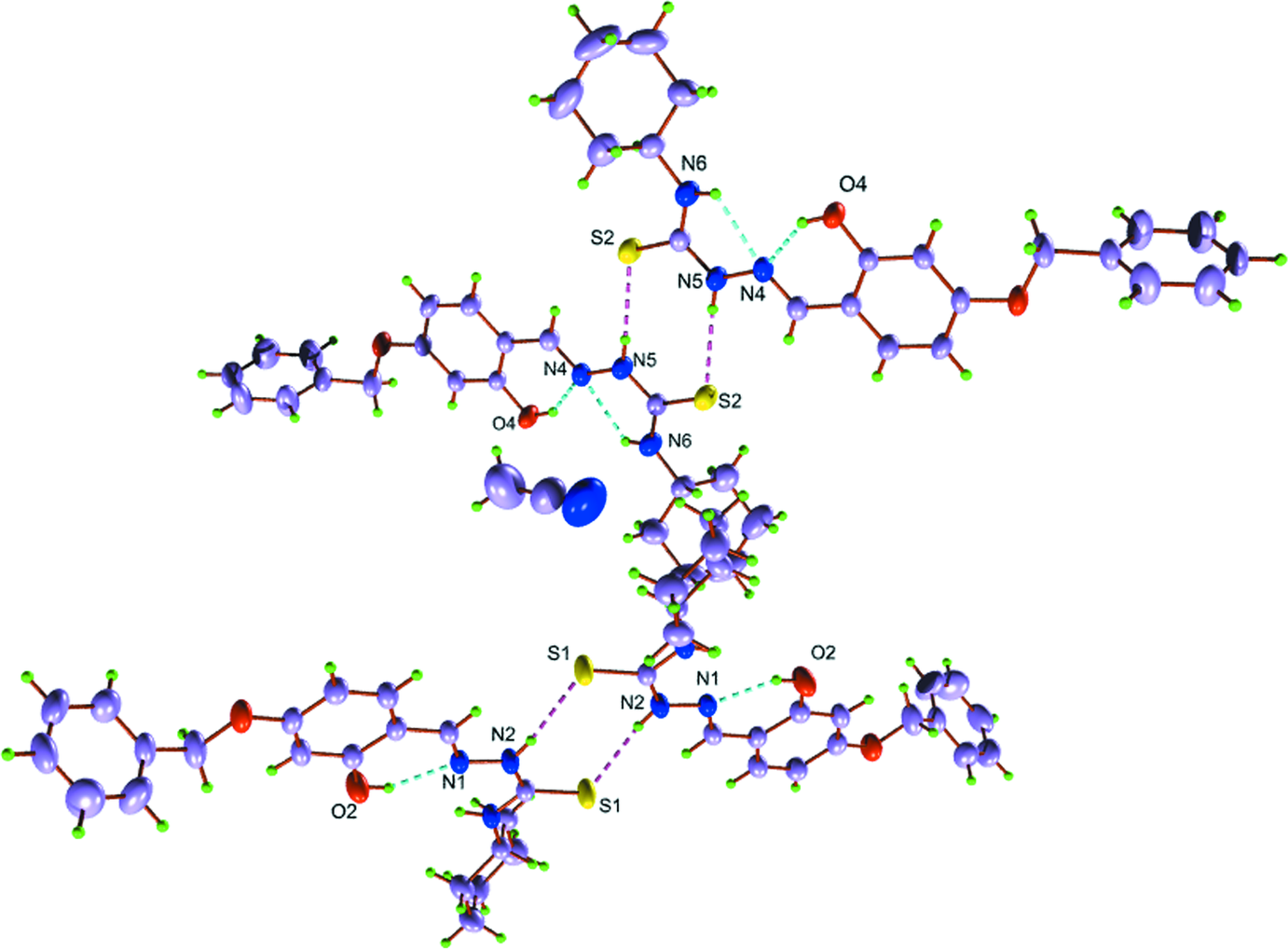

Supplement: Supplementary file 5 [file e-70-0o987-fig2.tif]

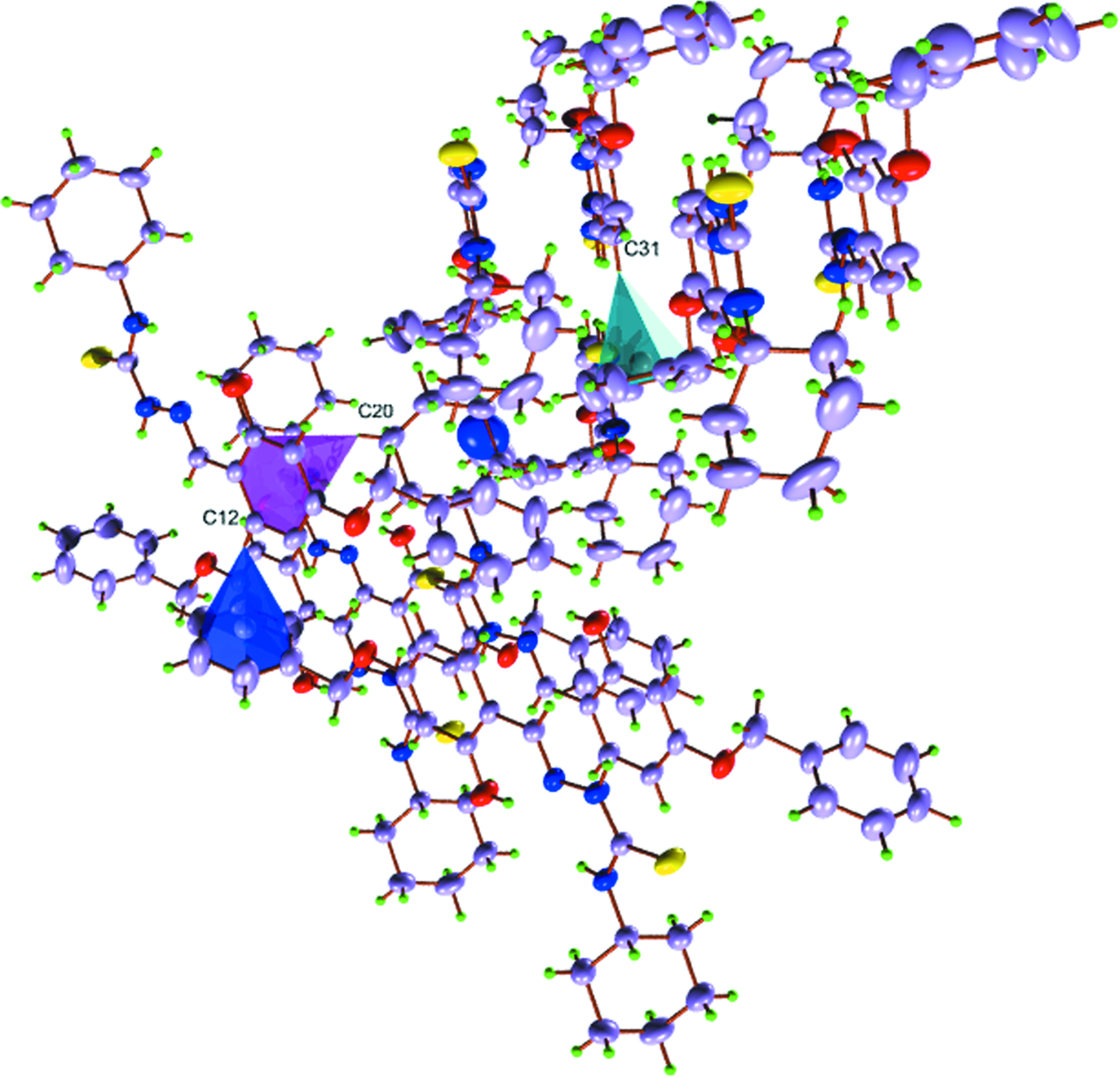

Supplement: Supplementary file 6 [file e-70-0o987-fig3.tif]

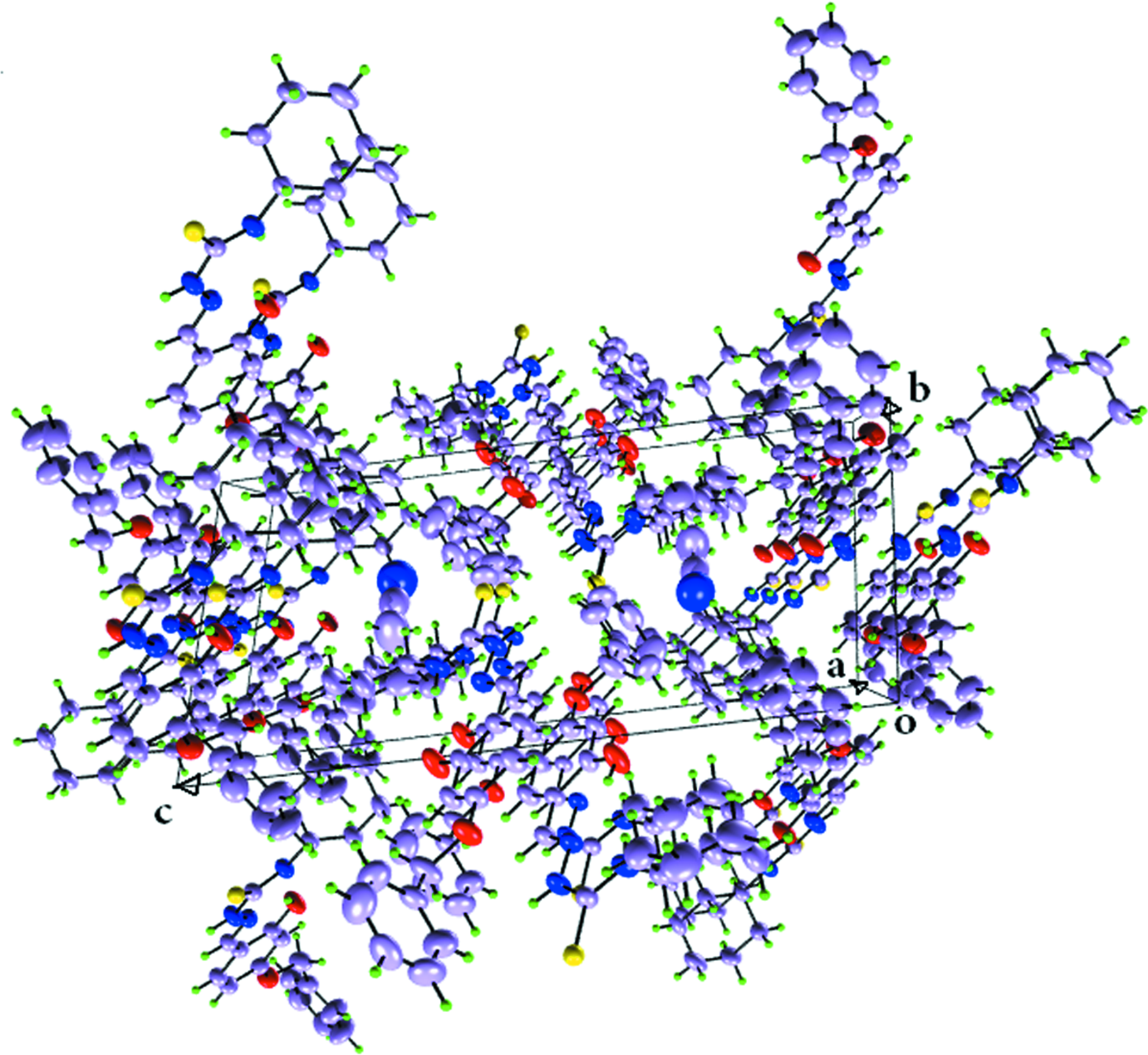

Supplement: Supplementary file 7 [file e-70-0o987-fig4.tif]
